# Circulating miRNAs in sepsis—A network under attack: An *in-silico* prediction of the potential existence of miRNA sponges in sepsis

**DOI:** 10.1371/journal.pone.0183334

**Published:** 2017-08-18

**Authors:** Catalin Vasilescu, Mihnea Dragomir, Mihai Tanase, Dana Giza, Raluca Purnichescu-Purtan, Meng Chen, Sai-Ching Jim Yeung, George A. Calin

**Affiliations:** 1 Department of Surgery, Fundeni Clinical Hospital, Bucharest, Romania; 2 Carol Davila University of Medicine and Pharmacy, Bucharest, Romania; 3 University Politehnica of Bucharest, Bucharest, Romania; 4 Department of Hematology, Fundeni Clinical Hospital, Bucharest, Romania; 5 Department of Mathematical Methods and Models, Faculty of Applied Sciences, Politehnica University of Bucharest, Bucharest, Romania; 6 Department of Experimental Therapeutics, The University of Texas MD Anderson Cancer Center, Houston, TX, United States of America; 7 Department of Endocrine Neoplasia and Hormonal Disorders, The University of Texas MD Anderson Cancer Center, Houston, TX, United States of America; 8 Department of Emergency Medicine, The University of Texas MD Anderson Cancer Center, Houston, TX, United States of America; 9 Center for RNA Interference and Non-coding RNAs, The University of Texas MD Anderson Cancer Center, Houston, TX, United States of America; Universitat de Barcelona, SPAIN

## Abstract

Biomarkers based on the molecular mechanism of sepsis are important for timely diagnosis and treatment. A large panel of small non-coding microRNAs was reported to modulate the immune response in sepsis but have not been tested in clinical practice. Large-scale identification of microRNA networks in sepsis might reveal a new biological mechanism that can be also targeted by gene therapy. Therefore, the main objective of this study is to perform a comparison of the miRNA network between septic patients and healthy controls. We used the previously measured levels of expression of 16 different circulating human and viral microRNAs in plasma from 99 septic patients and 53 healthy controls. We used three different computational methods to find correlations between the expressions of microRNAs and to build microRNA networks for the two categories, septic patients and healthy controls. We found that the microRNA network of the septic patients is significantly less connected when compared to miRNA network of the healthy controls (21 edges vs 52 edges, P < 0.0001). We hypothesize that several microRNAs (miR-16, miR-29a, miR-146, miR-155, and miR-182) are being sponged in sepsis explaining the loss of connection in the septic patient miRNA network. This was specific for sepsis, as it did not occur in other conditions characterized by an increased inflammatory response such as in post-surgery patients. Using several target prediction instruments, we predicted potential common sponges for the miRNA network in sepsis from several signaling pathways. Understanding the dynamics of miRNA network in sepsis is useful to explain the molecular pathophysiology of sepsis and for designing therapeutic strategies that target essential components of the immune response pathways.

## Introduction

Sepsis remains a major medical problem; each year 210,000 people die of sepsis in the United States, while diagnostic and therapeutic methods remain unreliable [1]. Crucial steps were made in understanding the pathophysiology of sepsis and new biomolecular mechanisms were unveiled [2–5]. This progress neither improves the diagnosis, nor the therapy, hence the outcome remains unchanged. However, the antibiotic therapy and the supportive care center (SCC) were the sole methods that increased the survival rate of septic patients in the last 30 years[6]. Recently, the results of DNA and RNA profiling identified microRNAs (miRNAs) as regulators of the immune response, with potentially translational implications in sepsis [7, 8]. MiRNAs are short 19–24 nucleotides transcripts that bind mRNA (targets), resulting in the inhibition of protein synthesis. Each miRNA can regulate multiple gene targets, while several miRNAs can target the same messenger RNA (mRNA). Their interaction occurs in the 3′-untranslated region (3′-UTR) of the target mRNA, causing translational repression or mRNA cleavage [9]. MiRNAs are released outside the cells into plasma and remain in circulation in a stable form, being resistant to endogenous and exogenous RNAs activity, acute pH and extreme temperature conditions [10, 11]. This makes miRNA expression pattern to be easily detected in the peripheral blood of septic patients [7].

A large panel of miRNAs was reported and experimentally validated to play a key role in modulating the immune response in sepsis [7, 12–15]. The vast majority of the studies published by now, focus solely on profiling the miRNA signature in plasma/blood cells; their interaction with various targets mRNA and on developing a potential biomarker that could differentiate sepsis from SIRS [16]. Many of these studies have looked only at miRNAs by their first-order relationships: what molecules regulate a miRNA and what mRNA transcripts are regulated by a miRNA [17, 18]. However, a new concept of gene regulation has recently emerged, suggesting that other RNA transcripts can have an important role in regulating miRNA’s availability within the cells, acting like natural miRNA decoys or sponges [19]. This adds another layer of complexity in studying the roles of miRNAs in sepsis, as it requires taking into account the “regulating the regulator” process. The study of this genome-scale regulating networks involving miRNAs and mRNAs for various protein coding genes including transcription factors is complex, as one has to describe the relationship between each of the components of this genomic network.

One proposed approach is to study the interaction between miRNAs using a network representation [20]. In general, molecular regulatory systems are represented as networks composed of nodes and edges, where the nodes can be genes, sequence elements, or molecules such as proteins, metabolites, RNAs and the edges (links) are the molecular interactions between the nodes [21, 22]. In a miRNA network, each node represents a circulating miRNA, while the edges represent the statistical dependence of their concentration (expression levels) in plasma. Finding the biological meaning of the edges is not an easy step, because of the incomplete information present in the molecular field of sepsis. Based on the mathematical model, any of the edges between miRNAs can be interpreted as targets for which the miRNAs can compete. This kind of approach was already used to build miRNA networks in cancer by Volinia et al [20]. Thus, we developed a model of miRNA networks for septic patients and studied the architecture and the dynamics of this network by comparing it with miRNA network in healthy controls. To prove the specificity of our approach, we studied the miRNA networks in a cohort of pre-surgical and postsurgical patients, as surgical trauma triggers a systemic inflammatory response similar to sepsis. Alterations of the sepsis miRNA network can cause significant changes in the outcome of the septic patients, as miRNAs regulate circuits that provide robustness to human protein-protein networks, signaling networks, transcript networks. Thus, it is important to analyze how perturbances in the components of the miRNA network can affect the functionality of these higher-level operating networks.

The purpose of this paper is to describe the changes in miRNA networks in sepsis. This approach is a first step in understanding the regulatory mechanism of miRNAs in the pathophysiology of sepsis. The role of miRNAs as sepsis biomarkers is highly debated [23]. We discuss the hypothesis that by describing miRNA networks in sepsis, we could establish a new diagnostic method to differentiate healthy controls and septic patients. This approach can also permit the identification of the key elements of the miRNA sepsis networks and investigate the changes cause. We believe that by describing the miRNA regulatory networks, new research paths in the field of sepsis biomarkers and even of sepsis therapy can be opened.

## Material and methods

### Patients and clinical samples

The data about miRNA level of expression in sepsis was obtained from our previous published study [8] where patients were recruited from two institutions, The University of Texas MD Anderson Cancer Center (MDACC), Houston, Texas and Fundeni Clinical Hospital (FCH), Bucharest, Romania. Sepsis was defined according to American College of Chest Physicians/Society of Critical Care Medicine [24]. The septic patients group was composed of 33 patients from FCH and 66 patients from MDACC. The FCH septic patients group had blood samples collected in the enrolment day at the hospital. Twelve patients out of 33 had a subsequent blood sample collected at day 7. In MDACC all blood samples were collected at the enrolment and for 15 of the patients also at day 7. The surgical patients group was recruited at FCH and included a group of 30 non-sepsis un-paired surgical patients; 19 samples were taken before surgery while 11 samples were taken on day 1 after surgery. Different from our previous study [8], we treated the sepsis cohort as a whole; we built the pre-surgical and post-surgical miRNA networks using only the unpaired surgical cohort and for the post-surgical miRNA network we used only the data collected on day 1 after surgery. The control group consisted of 53 healthy volunteers from MDACC, which were sampled once. Demographic data of the four patients groups are summarized in **S1 Table.** For this study, clinical data and blood samples were obtained after the participants gave the written informed consent, according to protocols approved by the FCH Ethics Committee and MDACC Institution Review Board. Additionally, the ethics committee of both institutions approved the methods employed in this study.

The networks have been constructed using the twelve miRNAs reported in our previous study [8], while additional four, also measured during the previous study, were not included in the final report. A total of 14 cellular miRNAs and 2 viral miRNAs (miR-K12-12* and miR-K12-10b) were measured by qRT-PCR in plasma samples of the four patient groups **(Table 1)**. RNA was extracted from 200 μl of plasma using a total RNA purification kit (NorgenBiotek, Thorold, ON, Canada) and eluted with 50 μl of elution solution. For the normalization of sample-to-sample variation in the RNA isolation step, the *Caenorhabditis elegans* cel-miR-39-3p, (mirVana® miRNA mimic, Applied Biosystems, Foster City, CA); was added (25 fmol of each in a total volume of 5 μL) to each denatured sample after mixing the plasma sample with lysis buffer. The RNA concentrations were measured using a NanoDrop ND-1000 spectrophotometer [8]. Afterwards RNA was retro-transcribed with SS III Reverse transcriptase to obtain cDNAs. Diluted cDNA (2.5 μL) was used as template in a qRT-PCR reaction with a total final volume of 5 μL. DNA amplification was performed using TaqMan primers specific for each of the miRNA together with SsoFast™ Probes Supermix (Bio-Rad Laboratories, Hercules, CA). The raw Ct values were normalized by cel-miR-39-3p (ΔCt = Ctgene–CtCel-miR) [8]. Data regarding four of the miRNAs used to build the networks were not published in our previous paper, since no significant changes in miRNA expression between sepsis patients and controls had been observed, in our previous study we treated the two sepsis groups separately (FCH sepsis group and MDACC sepsis group) [8]. These miRNAs are: miR-21, miR-29a, miR-155 and miR-223.

**Table 1:** Normalized plasma levels of the 16 miRNAs detected by qRT-PCR for the four patient groups (the values in the table are the normalized data of the number of cycles for each miRNA; *2* ^*-ΔCt*^, CT = the number of cycles). The two population data (MDACC and FCH septic patient groups) were pooled together as used for the network analysis presented in the manuscript.

	Sepsis (mean ± standard deviation)	Control (mean ± standard deviation)	Pre-surgery (mean ± standard deviation)	Post-surgery (mean ± standard deviation)
miR-146	0,4956 ± 1,2388	0,4045 ± 0,5935	0,03176 ± 0,03171	0,01627 ± 0,009304
miR-150	0,14136 ± 0,6691	0,03767 ± 0,03843	0,01693 ± 0,006835	0,01382 ± 0,007288
miR-155	0,03478 ± 0,1839	0,009113 ± 0,01409	0,0004117 ± 0,0002813	0,00153 ± 0,004051
miR-486	0,66113 ± 1,44524	0,3059 ± 0,2537	0,09814 ± 0,1091	0,1002 ± 0,1371
miR-16	11,5914 ± 28,3203	3,350 ± 3,300	0,7989 ± 0,9298	0,7834 ±± 1,404
miR-21	0,48337 ± 0,93555	0,1780 ± 0,2058	0,02005 ± 0,01575	0,01652 ± 0,01209
miR-29a	0,13165 ± 0,27103	0,01086 ± 0,008120	0,6451 ± 0,4109	0,6485 ± 0,2313
miR-182	0,00421 ± 0,0085	0,001466 ± 0,002985	0,0000032 ± 0.0000026	0,0000017 ± 0,0000017
miR-223	2,95182 ± 6,31515	1,267 ± 2,284	0,1020 ± 0,1733	0,05056 ± 0,04559
miR-23	0,01366 ± 0,03249	0,004970 ± 0,007263	0,0001189 ± 0,0001869	0,0000065 ± 0,0000087
miR-26a	1,41987 ± 3,39754	0,6085 ± 0,9975	0,006481 ± 0,006750	0,005517 ± 0,003665
miR-26b	0,9474 ± 2,24152	0,2568 ± 0,3975	0,006006 ± 0,006811	0,005034 ± 0,005601
miR-93	0,62634 ± 1,20462	0,1770 ± 0,1930	0,02736 ± 0,02943	0,02156 ± 0,03142
miR-342	0,04754 ± 0,09653	0,02378 ± 0,03320	0,0006272 ± 0,0004870	0,0004092 ± 0,0003300
KSHV-miR-k12- 10b	0,00898 ± 0,01759	0,0001075 ± 0,0001311	0,0001944 ± 0,0001925	0,000235 ± 0,0002743
KSHV-miR-k12- 12*	0,00105 ± 0,00237	0,0000073 ± 0,0001190	0.0000036 ± 0,0000032	0,0000051 ± 0,0000044

### Building the miRNA sepsis network

We used three different statistic approaches to generate the miRNA networks. The edges of the network are built using three different statistical methods, but the biological meaning of the edges was not established. *Ab initio*, it is important to understand that the nature of the miRNA networks is based on several assumptions.

The first approach to build a miRNA network in sepsis uses the **correlation coefficient method**. We used IBM SPSS statistics software for this analysis [25]. The correlation coefficient method was employed after the miRNA concentration and expression were measured in septic and control patients. Furthermore, a linear correlation between each element was searched for within a matrix of 16 elements. The existence of an edge between two nodes depends on the value of a correlation threshold. The value of this threshold establishes if an edge can exist between two nodes. Hence, the entire construction of the miRNA network depends on the value of this threshold. It is crucial to select the same threshold value for all the different patient groups. We select +0.8 as threshold value. This value is very high compared to other biological studies, where it is approximately 0.3–0.5 [26]. A lower threshold (e.g. 0.3) would have made the network difficult to analyze, because of the very high number of interconnections (edges), all nodes would have been connected in all the four networks constructed using this method. On the other hand, a higher threshold (e.g. 0.95) would have led to a network with isolated nodes, making our analysis impossible. An alternative would be to build a network with weighted connections, but in order to simplify the construction we chose this threshold. It is also important to understand that one condition to employ this method is to consider that the relation between the key components of the network is a linear one.

The second approach uses **cluster analysis to** establish the edges. The software suitable used for this second approach is the IBM SPSS statistics software hierarchical cluster analysis function [25, 27]. The software was set to group the nodes in different amounts of clusters. If there is just one cluster, all the nodes are connected to each other; if there are 16 clusters, when analyzing 16 elements, none of the nodes are connected. Inside a cluster all the nodes are connected to each other. It is also important to mention that this software can analyze the strength of the connection between two nodes. The strength of the connection is represented in distance. Therefore, in a given cluster all the nodes are connected, but the strength of the connections differs. For cluster analysis, it is important to choose the same number of clusters for the different patient groups. We built separate networks using two, four and six clusters. Finally, we chose to analyze only the four cluster networks, as this method provides the most suitable distance metric for the comparison between the networks of the four patients groups [28]. In order to construct a network where the different strengths of the connections are represented, it is necessary to choose several thresholds, which represent several strengths of connections. The same thresholds are employed for analyzing all the patient groups. We arranged the thresholds into six groups, each of them representing different connection strengths between the nodes and we displayed them in the miRNA networks. For more details, see **S2 Table**.

The third approach used is the **Bayesian network inference** that establishes the edges using Banjo software [20]. Building networks using the Bayesian method [29] is an approach widely used in studying biological networks. The Banjo software was adapted particularly for the purpose of this study. A Bayesian network is a graphical model for probabilistic relationships among a set of random variables. These relationships are encoded in the structure of a directed acyclic graph whose vertices (or nodes) are the random variables. The relationships between the variables are described by a joint probability distribution. A link from one node to another indicates that the “child” node is conditionally dependent on the “parent” node. Unlike many other network inference frameworks, Bayesian networks are capable of representing combinatorial, nonlinear, and stochastic relationships, such as those often found in biological systems. The Bayesian Network Inference with Java Objects (Banjo version 2.2.0) toolkit [30] was used for *static Bayesian Network* inference using specific settings. The static Bayesian network formalism means that the inference is made from steady-state expression data (the other formalism, dynamic Bayesian network can be used if time-series expression is available) [30]. The heuristic search method, *Simulated Annealing*, [30]that uses a stochastic decision mechanism wherein any network with a higher score is accepted with a probability, was employed. The construction of the network (edge change methods), *All Local Moves*, [30] produces a set of new networks to test using all possible single edge changes, with these networks then separately scored and compared to the current network according to the selected search algorithm. The metric and output, *in Banjo*, heuristic approaches (Simulated annealing or Greedy) are used to search the ‘network space' to find the network graph *G*. For each network structure explored, the parameters of the conditional probability density distribution are inferred and an overall network score is computed using the *Bayesian Dirichlet equivalence metric* (BDe) [30]. This score incorporate a penalty for complexity to guard against overfitting of data. The output network will be the one with the *higher score*. The discretization method used was *Quantile discretization* [30]. The nBestNetworks parameter was set to the value 1, representing the number of highest scoring networks to be tracked during a search stage. The setting eliminates the drawback of finding different equivalent subsets of the network during one search stage and improves the performance of the algorithm in terms of internal memory used and speed [30]. The Maximum time was set at 6 hours. Other parameter settings are the ones set by default.

Furthermore, we used Fischer’s exact test to verify if there is a statistically significant difference between the number of edges of the control group and the septic patient group. We employed this test for all the three methods used to generate networks. We used the Chi-square with Yates Correction to verify if significant differences exist between the overall distances of nodes of the control group versus the overall distance of nodes of the sepsis group. We used the same statistical tests for the pre-surgical and postsurgical miRNA networks.

### The miRNAs-mRNAs interaction: A simple predator-prey like mathematical model

The experimental data is insufficient to understand the behavior of the miRNAs in the different pathological states. The role of the miRNA remains uncertain and the mechanism of blocking the transcription is still controversial [31]. One possible way to understand the mechanism that lies behind the loss of edges is to use a mathematical model.

In order to test if competition is a possible cause of the changes in miRNA network in sepsis, we consider a simple mathematical model which is very similar to the classical Lotka-Volterra model [32]. For this reason, we will still call the species involved in the model “predators” and “preys/targets”. We define miRNAs as predators and mRNAs as preys. In this model, we consider the same constant rate of production both for miRNA and mRNA and a different reaction rate between miRNA and mRNA. After a predator consumes the prey, that predator cannot eat another prey so it disappears from the population of predators. We denote by X, Y the miRNA types (predators) and by A, B the mRNA types (preys) and we denote by x, y, a, b the corresponding concentrations of miRNA and mRNA, respectively.

We present below the mathematical model used in these simulations:

$$\frac{dx}{dt}=\rho -\alpha_{11}xa-\alpha_{12}xb$$

$$\frac{dy}{dt}=\rho -\alpha_{21}ya-\alpha_{22}yb$$

$$\frac{da}{dt}=\rho -\alpha_{11}xa-\alpha_{21}ya$$

$$\frac{db}{dt}=\rho -\alpha_{12}xb-\alpha_{22}yb$$

where:

x, y = the concentration of predators

a, b = the concentration of preys

*ρ* = the rate of production of predators and preys

*α*_11_ = the rate of reaction between the predator in X prey in A

*α*_12_ = the rate of reaction between the predator in X prey in B

*α*_21_ = the rate of reaction between the predator in Y prey in A

*α*_22_ = the rate of reaction between the predator in Y prey in B

We choose as initial conditions: x0 = 0.25 y0 = 0.35 a0 = 0.3 b0 = 0.2 and as parameters: *ρ* = 0.002 and *α*_11_, *α*_12_, *α*_21_, *α*_22_. These conditions do not influence the linear correlation between the nodes of the network. We choose these values only to create a simulation, these values do not reflect any biological reality. Choosing different initial values would not have changed the model [32].

### Target prediction method

We retrieved experimentally validated miRNA-mRNA interactions from several databases: miRTarBase (http://mirtarbase.mbc.nctu.edu.tw), TarBase (http://diana.imis.athena-innovation.gr/DianaTools/), miRWalk (http://www.umm.uni-heidelberg.de/apps/zmf/mirwalk/) and miRecords (http://c1.accurascience.com/miRecords/). We selected the experimentally validated targets for integrated pathway analysis with KEGG, Wikipathways, Reactome, BioCarta, Panther using Enrichr bioinformatics resources (http://amp.pharm.mssm.edu/Enrichr/). The p-value less than 0.05 was considered significant and selected.

## Results

**Fig 1:**
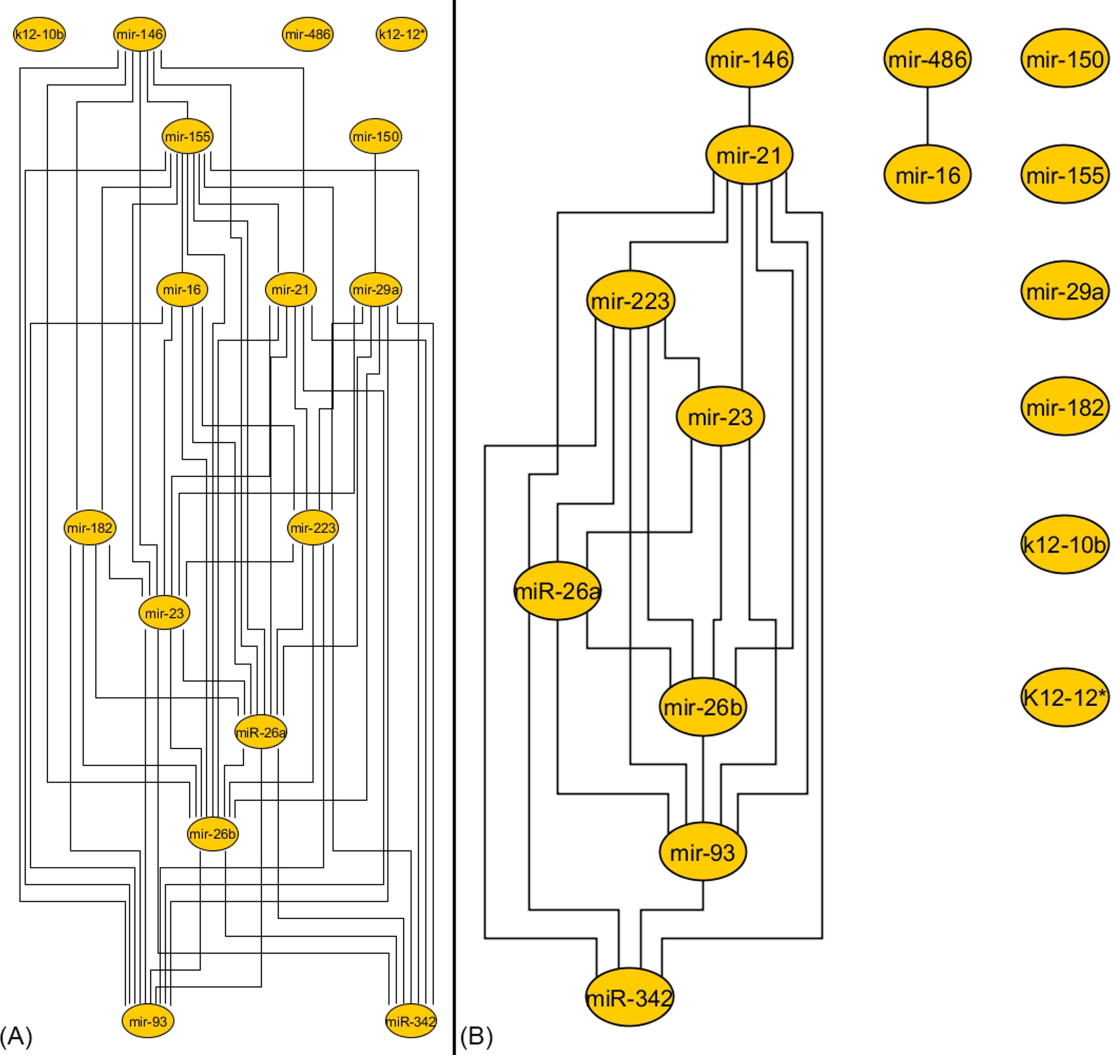
The miRNA networks for control and septic patients built using the correlation coefficient method. The control patient network is presented on the left side (A), the nodes of this network being highly interconnected through 52 edges. On the right side (B) is the sepsis patient network, the number of edges decreasing to 21, a decrease of 59.62% (statistically significant, P < 0.0001). Inside the sepsis network 6 nodes are completely isolated, which are weakly connected in the control group network, with one exception, miR-155. The two viral miRNAs, miR-K12-12* (k-12-12*) and miR-K12-10b (k12-10b), are not connected to the main miRNA network in the control patient or in the septic patient network.

**Fig 2:**
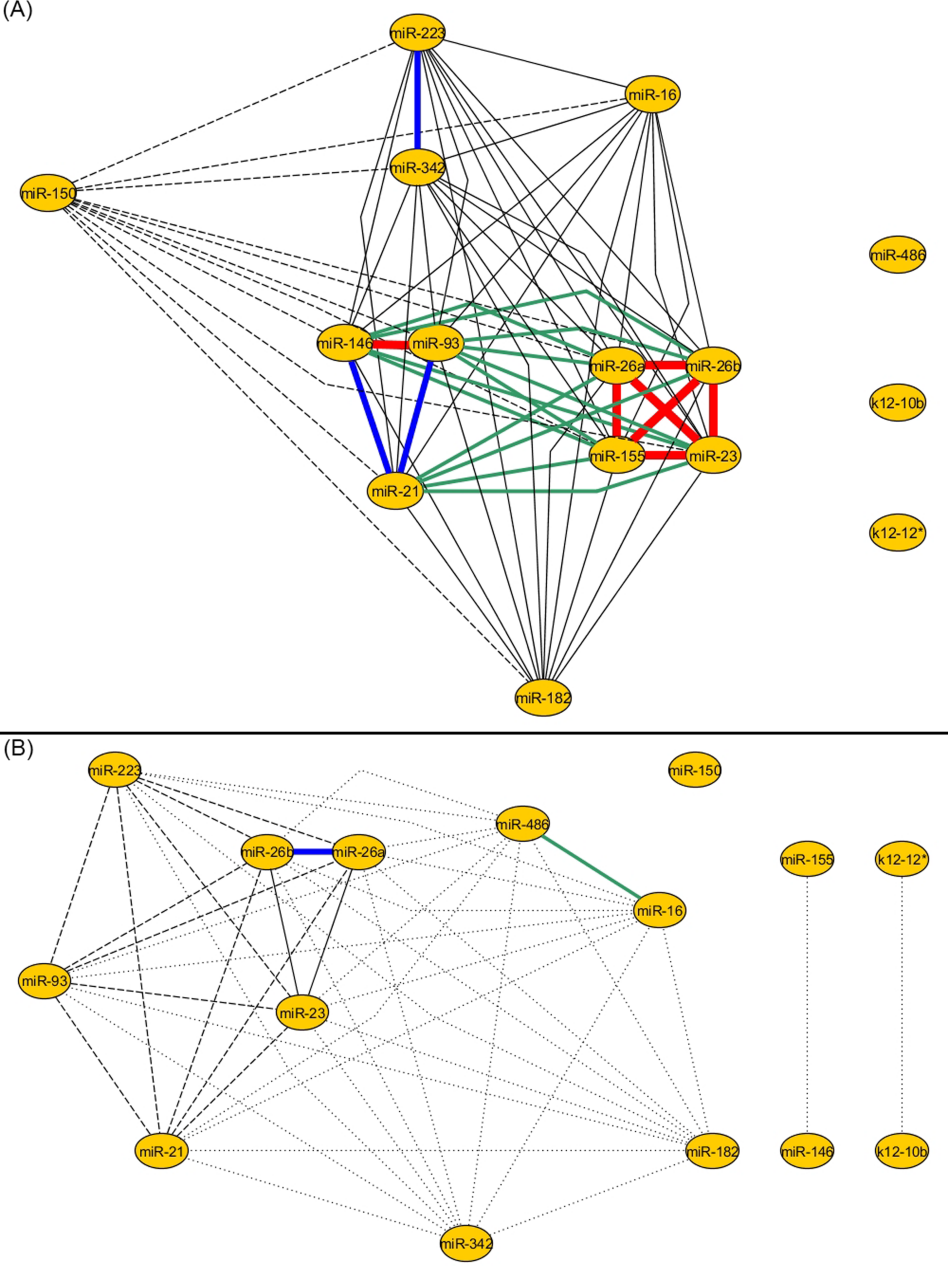
The miRNA networks for control and septic patients built using cluster analysis. The number of edges decreases between the control (A) and septic network (B), from 66 to 47; this means a decrease of 28.79% (statistically significant, P = 0.0125, the strength of connection between two miRNAs is represented using a color legend, for more details see **S2 Table**). Furthermore, the strength of the connections decreases significantly, the total distance between the nodes of the control group is 1595 and the total distance between the nodes of the sepsis group is 7370, P = 0.0001. Again, the viral miRNAs are isolated from the control and septic patient network.

**Fig 3:**
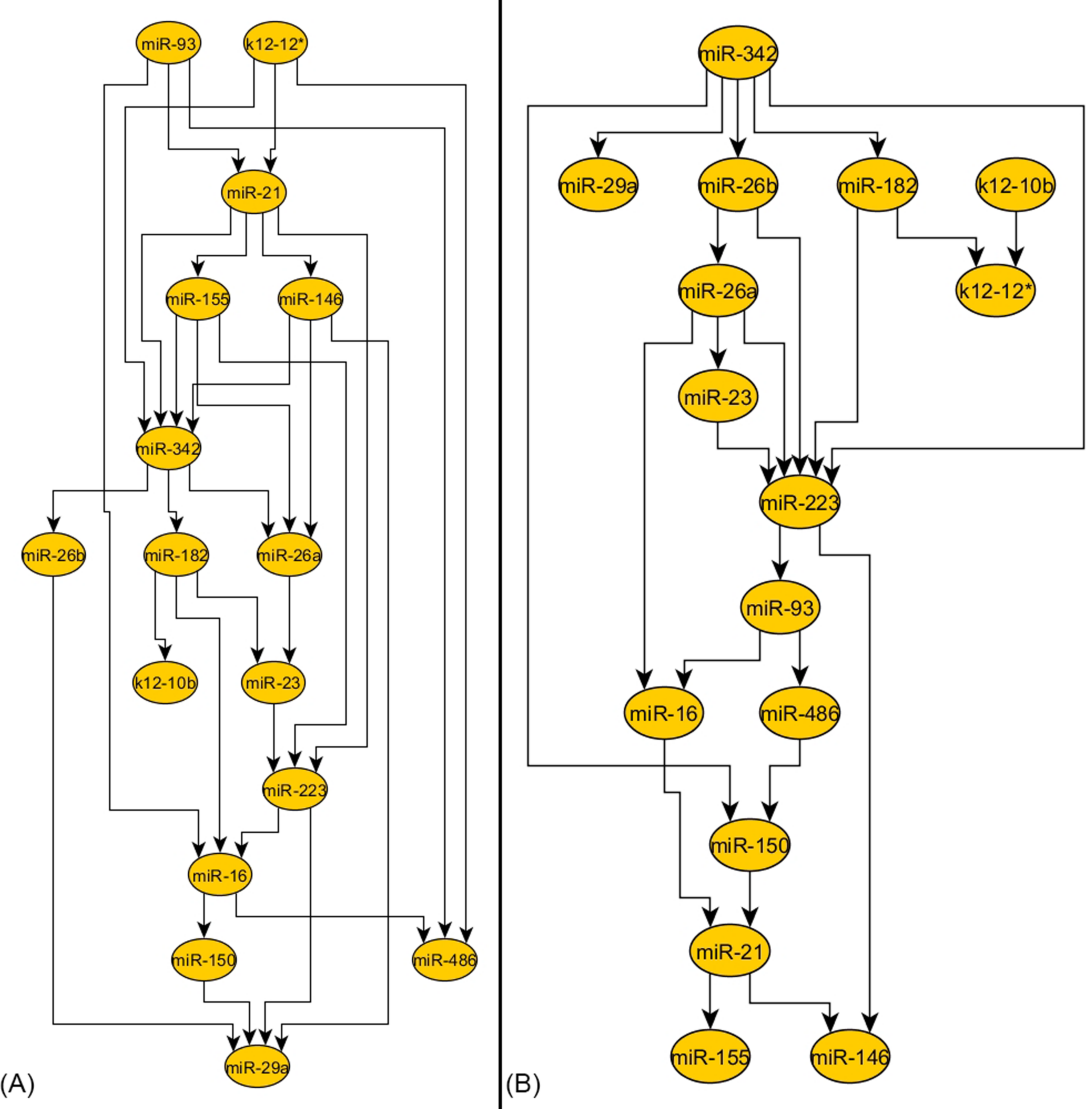
The miRNA networks for control and septic patients built using Bayesian inference. On the left side the control group network is depicted (A) and, on the right side the septic patient network is shown (B). The number of edges decreases between the two networks, from 30 edges in the control group to 23 edges in the septic group, which means a decrease of 23.33% (not statistically significant, P = 0.3505). In both, control and septic patient network, the same miRNAs remain very well connected (miR-21, miR-223 and miR-342). The viral miRNAs are connected to the networks in both cases, but their connection is weak.

By comparing miRNA networks in healthy controls to miRNA networks in septic patients, we observed that the septic miRNA networks are less connected (i.e. have fewer edges) (**Figs 1–3**). All three statistical methods employed confirm our observation.

### 1. Lower connectivity of miRNA network in septic patients: The correlation coefficient method

Using the correlation coefficient method, we observed that the number of edges decreases significantly from 52 in the control patient network to 21 edges in the septic patient network (P < 0.0001), which means a decrease of about 60% **(Fig 1).** The two viral miRNAs, miR-K12-12* (k-12-12*) and miR-K12-10b (k12-10b), are not connected to the main miRNA network in the control patient or in the septic patient network. Furthermore, miR-486 is not connected to the miRNA network in the control group. In the control patient network, miR-23, miR-26a and miR-26b are strongly connected each through 11 edges; miR-93 and miR-155 are also strong connected to 10 other nodes through 10 edges. Contrary, the septic patient network reveals that 6 miRNAs are completely isolated. These particular miRNAs are weakly connected in the control group, 6 edges or less, with one notable exception, miR-155. MiR-155 is connected to 10 nodes in the control group and loses all its connections in the septic patient network. In the septic patient group, we observed there are two different networks, one built out of 8 miRNAs and the other built only out of 2 miRNAs connected by one edge. Interestingly, miR-486 is part of this small network.

### 2. Lower connectivity of miRNA network in septic patients: The cluster analysis

Using the hierarchical cluster analysis method, we observed the same phenomenon as revealed by the correlation coefficient analysis: a loss of complexity between the control group network and the septic patient group. The number of edges decreases significantly from 66 in the control group to 47 in the septic group (P = 0.0125), which means a decrease of 28.79% (Fig 2).

These two networks are each built out of 15 miRNAs. We noticed that the strength (the distance between miRNA nodes) of the connections decreases significantly between the control group and septic group (total distance between nodes of the control group is 1595 and the total distance between the nodes of the sepsis group is 7370, P = 0.0001). The same miRNAs are separated from the main network as first identified by the correlation coefficient method. The miRNAs isolated from the network are the two viral miRNAs and miR-486 for the control group and miR-150 and miR-155 for the sepsis group. Furthermore, the nodes with the higher number of connections in the correlation coefficient analysis are the ones with the most powerful connections in the cluster analysis networks. These nodes are represented by miR-26a, miR-26b, miR-23, miR-93 miR-146 and miR-155 for the control group. The same miRNAs found to be well connected in the septic patient network after using the correlation coefficient method were found to have a high number of connections when using the cluster analysis method, with one exception that is represented by miR-155. The behavior of miR-486 is also similar between the two methods (cluster analysis and correlation coefficient analysis): miR-486 is isolated from the control group network, but is a part of the network in the septic group.

### 3. Lower connectivity of miRNA networks in septic patients: The Bayesian method

Using the Bayesian method, the number of edges was found to decrease from 30 in the control group, to 23 in the septic patient group, this difference is not statistically significant (P = 0.3505). This represents a decrease of 23.33% **(Fig 3).** In both control and septic patient network, the same miRNAs remain very well connected. The two viral miRNAs are poorly connected in control and sepsis networks, similar to the networks built using the other two methods.

### 4. MiRNA networks in pre-surgical and post-surgical patients

In order to demonstrate the specificity of our findings we employed the same methods to build the miRNA networks for pre-surgical and postsurgical patient groups, as surgical trauma triggers a systemic immune response similar to the one that occurs in sepsis. In contrast with septic patients, the number of edges increased in post-surgery group compared to pre-surgery group, when using the correlation coefficient method and the Bayesian method (from 5 to 28, P = 0.0001 and from 20 to 29, P = 0.1999). In the case of cluster analysis, the number of edges decrease, from 49 to 37, P = 0.0763, which is not statistically significant, but the total distance significantly decreased between the networks, from 1138 to 188 (P = 0.0001), opposite to the observations made for the control and septic patient miRNA networks. These networks are depicted in **S1–S3 Figs**. Thus, not any type of stress (in our case surgical trauma) that triggers a systemic immune response causes the loss of connectivity in miRNA network.

### 5. The miRNA network is “sponged” in sepsis

**Fig 4:**
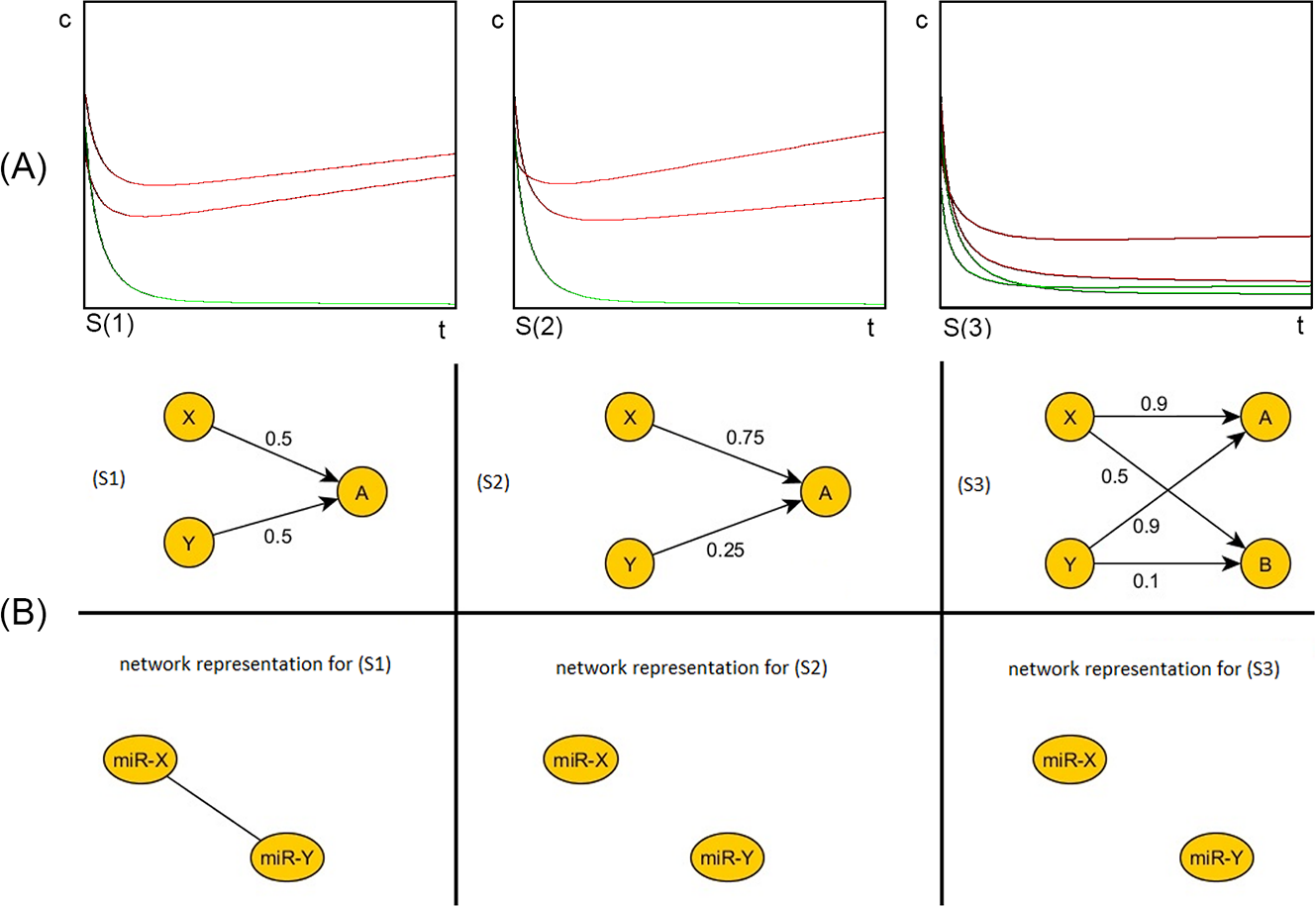
**The simulation of the mathematical model (panel A) and the network representation of three possible scenarios (panel B).** On the upper row the dynamics of the three scenarios: (S1), (S2), (S3) are represented (panel A). Legend: red = predators, green = preys, t = time, c = concentration. The horizontal axis represents time (t) and the vertical axis the concentration (c) of the predators and preys. By simulating the model for the three scenarios, we obtained the three set of data visualized in this figure: (S1) represents a connected network, where the upper red line is the concentration of the predator Y, the lower red line represents the concentration of the second predator X and the green line is the concentration of the the prey A; (S2) and (S3) represent disconnected networks. In the second scenario (S2) the initial higher concentration of Y decreases rapidly because of the low reaction rate (0.25) and the initial lower red line (the concentration of X) increases rapidly because of the high reaction rate (0.75), the lower green line represents the prey (A). In the third scenario the initial concentrations are the same: the upper red line is the concentration of Y which decreases rapidly because of the low reaction rates (0.9 and 0.1), the lower red line is the concentration of X, which will increase, because of the high reaction rates (0.9 and 0.5), the upper green line is the prey A and a new prey appears (B)–the lower green line. The concentration of A will decrease more rapidly than that of B, because of the rising concentration of the predator X, who has a high reaction rate for A and B (0.9, respectively 0.5). On the middle and lower rows three corresponding network representations are shown (panel B). The first column of the B panel represents a scenario (S1) that could reflect the mechanisms in the control group. In this case, we have two miRNAs (X and Y) competing for one common target A. The numeric value represents the reaction rates between the miRNAs and their target. In the network representation of this scenario, the two miRNAs are connected. In the second column of panel B, the scenario S2 is depicted and its network representation. In this case, the reaction rate between X and A (0.75), is different from the reaction rate between Y and A (0.25). In the corresponding network representation, the miRNAs are no longer connected. Thus, a different reaction rate between the miRNAs and a common target could be the underling mechanism that could explain the loss of edges in sepsis. The third column of panel B represents the S3 scenario. In this scenario, a new target appears (B). The two miRNAs X and Y have the same reaction rate for the first target (A), but very different reaction rates for the new target (B), 0.1 and 0.5, respectively. In the corresponding network representation, the two miRNAs are no longer connected. The S3 scenario represents, as well, a possible underling mechanism that explains the loss of connections observed in sepsis.

We considered 3 basic scenarios and we obtained the following dynamics depicted in **Fig 4** panel A and the following linear correlation networks with a simple 2 nodes situation. In the scenario S1, where two predators X and Y compete for one prey A, the two predators have the same reaction rate with the prey. In the scenario S2, the two predators X and Y compete for one prey A and the reaction rates with the prey (X-A and Y-A) are different. Finally, in the scenario S3, where the two predators X and Y compete for two preys A and B, the reaction rates with X-A and Y-A are the same and the reaction rates for X-B and Y-B are very different. These scenarios are depicted in **Fig 4** panel B.

We observe that in the first scenario (S1) we have a connected network showing that the concentration of the two predators is linearly correlated as an effect of competition over the same target. In scenarios (S2) and (S3), the network is disconnected and the linear correlation decreases with the increasing difference of the reaction rates between the predators and the prey or the presence of a new prey with a higher reaction rate with one of the predators. The networks corresponding to the scenarios are shown in **Fig 4** panel B. Therefore, we consider that the correlation between two predators is a consequence of the competition over a common target. Consequently, a prey which is the subject of competition for the two predators increases the linear correlation between the concentrations of the predators.

### 6. Sepsis–a miRNA network under attack

**Fig 5:**
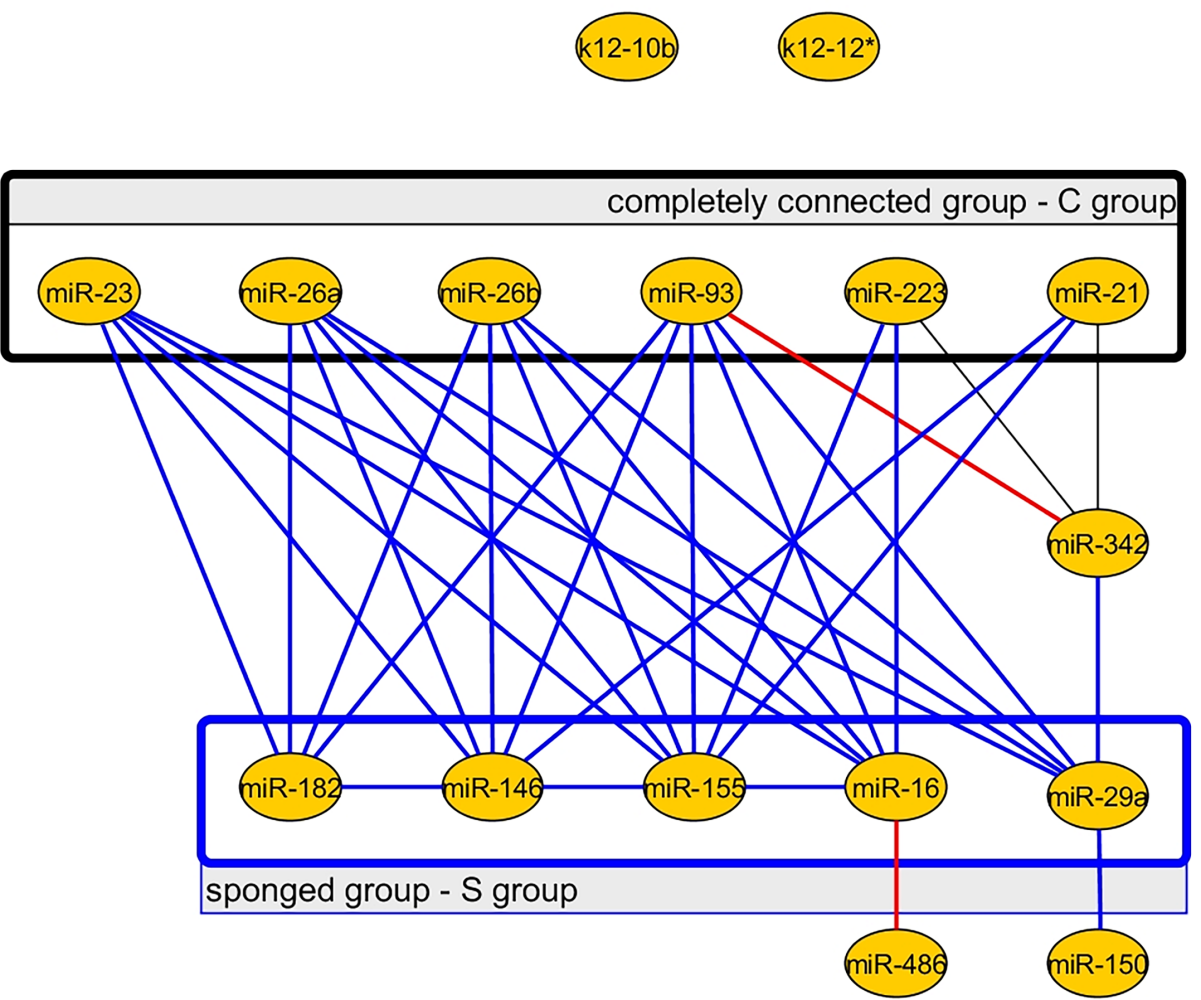
The miRNA network representing all the edges that remain both in control and sepsis, newly appear and disappear in sepsis, when using the correlation coefficient method. The edges that remain both in control and sepsis are represented by the black lines, the edges that disappear in sepsis are represented by blue lines and the two new edges that appear in sepsis are represented by red lines. New edges appear between miR-93 and miR-342 and between miR-16 and miR-486. The C group is a completely connected group both in control and sepsis (black rectangle). The C group is composed of six miRNAs–miR-23, miR-26a, miR-26b, miR-93, miR-223 and miR-21. The S group (blue rectangle) is a group of miRNAs that is highly connected in the control patient network, but loses all of its connections in sepsis. In sepsis, the internal connections of the S group (blue rectangle) and the connections between the S and C groups are lost.

In order to have a closer look on the change of connectivity between control patient and septic patient miRNA networks, we generated a new miRNA network representation (**Fig 5**). This network contains all the edges that are lost in sepsis (blue edges), newly appeared in sepsis (red edges) and those that are present in both biological states (black edges). We noticed that miR-155 loses more edges in sepsis when compared to the other miRNAs in the network. Also, we observed that only two new edges appear (red edges), an edge that connects miR-342 with miR-93 and an edge that connects miR-16 with miR-486. The viral miRNAs, miR-K12-12*(k12-12*) and miR-K12-10b (k12-10b) are both disconnected from the other miRNAs in the sepsis network and the control network.

Furthermore, we separated the miRNAs in two different groups based on their connectivity (**Fig 5**). The C group (connected group) was considered the completely connected group; meaning that all the miRNAs of this group were connected with each other and remained connected in both biological states (black edges and black rectangle). In contrast, the S group (sponged group) was identified to be a very well connected in controls, but lost all of its connections in sepsis (blue edges and blue rectangle). Furthermore, we observed that all the connections between the S group and the C group disappeared in sepsis (blue edges). To better illustrate our findings, we applied the principle of predator-prey like mathematical model to our groups of miRNAs, S = {miR-182, miR-146, miR-155, miR-16, miR-29a} and C = {miR-23, miR-26a, miR-26b, miR-93, miR-223, miR-21}.

Moreover, we retrieved experimentally validated data on the miRNA-mRNA interactions from several databases to identify which targets are in common for all five miRNAs in S group, that may act as the sponge and be responsible for the architectural changes of the S group network. In order to determine that these targets are specific to the S group, we predicted the common targets of the C group and compared these targets with those of the S group. As shown in Table 2, the known targets common for all the miRNAs from a group are specific: for group S, there are eight common targets, ATP13A3, CDKN1A, GSK3B, RAPH1, TET3, TP53INP1 and TSPAN14, while for the group C there are three different targets, including AGO2, MDM2 and SP1 (**Table 2**). Finally, when we were counting for the common targets for (n-1, where n is the maximum of miRNAs from each group) miRNAs from the two groups, we identified a set of different signaling pathways for groups S and C (**S3 Table**). These results point at the potential existence of sponging mRNAs specific for group S miRNAs.

**Table 2:** Common experimentally proven targets for all miRNAs from S group and C group, and examples of sepsis related references of this genes. (N/A, not available).

miRNAs	Gene Symbol	NCBI gene name	Examples of sepsis-related references
Group S (miR-182, miR-146, miR-155, miR-16, miR-29a).	ATP13A3	ATPase 13A3	N/A
	CDKN1A	cyclin dependent kinase inhibitor 1A	[33]
	GSK3B	glycogen synthase kinase 3 beta	[34]
	RAPH1	Ras association (RalGDS/AF-6) and pleckstrin homology domains 1	N/A
	SLC38A1	solute carrier family 38 member 1	N/A
	TET3	tet methylcytosine dioxygenase 3	N/A
	TP53INP1	tumor protein p53 inducible nuclear protein 1	N/A
	TSPAN14	tetraspanin 14	N/A
Group C (miR-21, miR-23, miR-26a, miR-26b, miR-93, miR-223).	AGO2	argonaute 2, RISC catalytic component	[35]
	MDM2	MDM2 proto-oncogene	[36]
	SP1	Sp1 transcription factor	[37]

## Discussion

The construction and analysis of miRNA networks in sepsis is a completely new idea and an uncharted territory. To our knowledge there is no comprehensive study published by now on the miRNA network in sepsis. As miRNAs have been proposed as possible new diagnostic and prognostic tools, the implications of sepsis miRNA network and its dynamics should be considered for extensive research.

Circulating, cell-free miRNAs, due to their high stability in plasma and serum, can be considered a new class of molecular biomarkers that can be used in diagnosis, prognosis and monitoring of treatment response [8]. In diseased tissues, like solid cancer, miRNAs changes are reflected in the circulation [38]. Particular expression patterns in plasma and in serum for various diseases were identified [39–41]. Because each miRNA can control the translation of tens or hundreds of mRNAs and each mRNA is controlled by several miRNAs [42], it is difficult to assess one miRNA specificity for a given disease. For the non-neoplastic disease biomarkers, only one subset of reported miRNAs has specificity for a particular disease. This is also the case of sepsis, where several miRNAs involved in immune response regulation have been proposed as a miRNA signature in sepsis; however none has been shown to be specific for sepsis diagnostic and prognosis [43, 44]. Halushka et al underlined the fact that most biomarkers are considered either non-specific or uninterpretable since there are still significant limitations in our knowledge about miRNAs expression [44]. Although several thousand human miRNAs were identified by now, only a small proportion of them are sufficiently abundant to exert a quantifiable posttranslational regulation [45]. Moreover, quantification of miRNA repression effect on proteins, transcription factors and other RNA transcripts is important. Therefore, a comprehensive view on the gene regulation network that includes proteins, transcription factors, RNA transcripts (mRNA) and miRNAs is important. However, the construction of this extensive network is beyond current biological knowledge and available analysis methods. We limited our research on the indirect interactions between miRNAs. This can be done by using the network theory, as it is one of the most common methods to describe complex biological systems [46]. We used this approach in order to build the miRNA network in septic patients and compare it to miRNA network in healthy controls.

One first observation we made was that the miRNA network in sepsis is less connected. The miRNAs seem to be “sponged”, this causing a decrease in the number of edges. The sponge theory is not new; several researchers reported that mRNA and other types of non-coding RNA can sequester miRNAs; hence, the activity of these miRNAs is altered [19, 47–52].

We used the predator-prey like mathematical model to analyze these findings. We noticed that a group of mRNAs that act like a sponge may be responsible for the disappearance of the edges that connect the miRNAs inside the group. One possible explanation is that the miRNAs within the S group are “sponged” by mRNAs that are overexpressed in sepsis. This hypothesis is supported by the changing in dynamics of the system as described by the mathematical model. A scenario represents the dynamics by using our mathematical model, and the correlation network obtained by using the linear correlation analysis for a given affinities setup. We hypothesize that the 2x1 and 2x2 node scenarios studied here capture the core mechanism that causes the gain and loss of links between nodes in the network. In this view, the global change of the network will be a composition/superposition of many instances of these scenarios, the different combinations of nodes that are in such types of relation. In details, the S3 scenario presented in supporting Fig 4 is similar to “the sponging phenomenon” of the S group, and the competition inside the S group and between the S group and C group disappear. In sepsis, the group S is modified by a common mRNA/non-coding RNAs (ncRNAs) or multiple mRNAs/ncRNAs that is/are up-regulated. The main conditions for the “sponging phenomenon” to take place is that: i) the sponge has a very high reaction rate to the miRNAs of the S group, and ii) mRNA(s) is (are) available in high concentration. The high reaction rate of the sponge(s) is responsible for the loss of competition between S and C groups, explaining the disappearance of edges connecting miRNAs from S group with miRNAs from C group (edges outgoing from the lower rectangle in **Fig 5**). The high concentration of the sponge also explains the disappearance of the edges connecting the miRNAs inside the S group (edges inside the lower rectangle in **Fig 5**).

We also observed that in sepsis, new edges can appear. In the case of miR-342 –miR-93 one may presume that before sponging, this edge was not present because of the lower reaction rates between these two miRNAs and the common target compared to the significantly higher reaction rate between the miRNAs of group S with this specific target. After sponging, despite the lower reaction rates, the target becomes more available for miR-342 and miR-93, thus increasing their competition over it. This was reflected by the appearance of a new edge between miR-342 and miR-93 (red line in Fig 4).

In the case of miR-486 –miR-16 edge, we presume that in sepsis, both miR-486 and miR-16 react with a sponge. Their competition over the sponge can be explained by a high concentration of these two miRNAs, comparable to the concentration of the sponge.

Several investigations were conducted to understand how miRNAs regulate other biological networks. We analyzed the targets that are common for the S group and we identified eight genes: ATP13A3, CDKN1A, GSK3B, RAPH1, TET3, TP53INP1 and TSPAN14. Very interestingly, several of these genes were already linked with sepsis in various models, further supporting the approach that we presented in this paper (**Table 2**). These genes are different from the common targets of the C group (three common genes: AGO2, MDM2 and SP1). Hence, we can consider that one/several of the targets specific for the S group are up-regulated in sepsis and determine the loss of connectivity of the miRNAs of this group, by sponging the miRNAs. Finally, we identified the signaling pathways specific for the target genes of the S group and C group. Most of the signaling pathways specific for the targets differ between the S groups and C group.

Our study has the following limitations. Firstly, it is important to understand that the networks we built are based only on the expression level of 16 miRNAs and three different statistical methods. This approach is based on several assumptions. In the case of the correlation coefficient method, the two assumptions are: (1) that the relation between the nodes is linear (if this relation is not linear, then a much more complex procedure must be used, e.g.—Chebyshev polynomials, which may be a matter of further research), and (2) the use of a 0.8 threshold. In the case of the cluster analysis other two assumptions apply: (1) we used 4 clusters for all the networks we depicted, and (2) the six thresholds used to construct the networks (see S1 Table). In the case of steady-state data (static Bayesian network), Banjo—as other Bayesian network algorithms—is not suitable to infer networks involving cycles (e.g. feedback or feed forward loops) [30]. In addition, we are aware that networks constructed only by using the expressions of miRNAs, only partially represent the biological reality, a complete regulatory network being composed of miRNAs, mRNAs, proteins, ncRNAs and transcription factors.

Secondly, we explained the loss of the edges in the septic network assuming an hypothesis which is only partially demonstrated–the sponge theory [45], that needs further validation. There are other theories that could explain the loss of edges (changes of the miRNA expression in plasma). For example, we could presume that the mechanism that confers stability to circulating miRNAs in controls is altered in sepsis [53], affecting only the miRNAs of the S group. MiRNAs travel in plasma co-fractionated with protein complexes, inside exosomes and microvesicles. In sepsis one of this mechanism could be altered. It is highly possible that a biological meaning exists between to interconnected nodes, but the true explanation remains unknown. Future *in vivo* research could confirm these theories.

Thirdly, we used only *in-silico* methods to predict the possible sponges of the S group, which is a very limited approach. It will be our next step to confirm the existence of such sponges in biological samples from septic patients. We also determined the specific pathways of the identified sponges, this observation also need additional *in vivo* studies for confirmation.

Fourthly, regarding the septic patient group, the pre-surgical group and the post-surgical group, considering that most of these patients are affected by cancer, it is important to mention that previous studies [20] reported modified miRNA networks in cancer. It is uncertain which of the observed changes are sepsis/SIRS specific and which can be attributed to the underlying oncologic disease.

In spite of the above-mentioned limitations, our observation is confirmed by the three different methods; in sepsis the number of edges decreases and this observation reflects a biological phenomenon. Our observations regarding the cause that determines the loss of edges inside the sepsis network is theoretical, but necessary to direct future research. Additional research is needed to construct more complex networks containing a higher number of miRNAs and different molecules (sponges). Nevertheless, the field of miRNA biomarkers in sepsis is very debated [23], and we consider that by confirming our observation (loss of edges in miRNA sepsis networks) on a higher scale, this data can be used to diagnose sepsis.

## Conclusions

Alterations and malfunctions of the sepsis miRNA network might cause significant changes in the outcome of the septic patients, as miRNAs regulate circuits that provide robustness to human protein-protein networks, signaling networks, transcript networks etc. Identifying the specific miRNA pattern in sepsis could profoundly influence the design of clinical studies involving miRNA-based diagnostic and therapeutic strategies.

The study of miRNA network in septic patients may also provide further insights about how higher-level biological processes, such as the immune response in sepsis, are made less robust. One of the greatest advantages of understanding more about the dynamics of miRNA network in sepsis translates into the ability to design future diagnostic and therapeutic strategies that target essential components of the immune response. Therefore, employing miRNAs mimics or antisense-RNA inhibitors to manipulate essential nodes or “sponges” of the regulatory network might represent potential new therapeutic avenues in septic patients.

## Supporting information

S1 Table Demographic data of the four patient groups. (PDF)

S2 Table The six different thresholds used to build the miRNA networks using the cluster analysis method. (PDF)

S3 Table The signaling pathways for the microRNAs targets of the S and C groups. Only signaling pathways highly significant at P < 0.001 are included. Marked in colors are the few common pathways between the two groups of miRNAs, S and C.
(XLSX)

S1 Fig The pre-surgical and postsurgical miRNA networks built using the correlation coefficient method. On the left side the pre-surgical miRNA network is represented (A) which contains only 5 edges, on the right side the postsurgical miRNA network is depicted (B), which contains 28 edges. After the surgical procedure the number of edges significantly increases from 5 to 28 (P = 0.0001), this is on increase of 82.14%. All the existing edges from the pre-surgical group are also present in the post-surgical group.
(TIF)

S2 Fig The pre-surgical and postsurgical miRNA networks built using cluster analysis method. In the upper part of the figure the pre-surgical miRNA network is depicted (A), which is built out of 4 clusters and a total of 49 edges. In the lower part of the figure the post-surgical miRNA network is depicted (B), consisting of 4 clusters and 37 edges. In this case the number of edges decreases, from 49 to 37 (P = 0.0763 –not statistically significant), this is a decrease of 24.48%. This observation is similar to the data obtained for the control and septic patient networks, where the number of edges also decreases, but in the case of cluster analysis, much more meaningful is the distance between the edges, which clearly increases between the pre-surgical network and the post-surgical network. In the case of the control and septic patient network the distance decreases between the nodes of the control and septic patient miRNA networks. The total distance of the pre-surgical miRNA network is 1138 and the total distance of the post-surgical miRNA network is 188 (P = 0.0001). This observation is opposite to what we observed in the case of control and sepsis miRNA networks, where the distance decreases.
(TIF)

S3 Fig The pre-surgical and postsurgical miRNA networks built using Bayesian interference. On the left side the pre-surgical miRNA network is represented (A), composed of 16 nodes and 16 edges, on the right side the postsurgical miRNA network is depicted (B) composed of 16 nodes and 29 edges. When using this method, the number of edges increases between the two networks, from 20 to 29, an increase of 31.03%, this difference is not statistically significant (P = 0.1999). This observation is opposite to what we observed in the case of control and sepsis miRNA networks built using the Bayesian method, where the number of edges decreases.
(TIF)
