# Supplementary material for: Circulating miRNAs in sepsis—A network under attack: An in-silico prediction of the potential existence of miRNA sponges in sepsis
Source: PLoS One. 2017 Aug 18;12(8):e0183334. doi: 10.1371/journal.pone.0183334 (PMC5562310; doi:10.1371/journal.pone.0183334)
Supplement: S1 Table — (PDF) [file pone.0183334.s001.pdf]

Cohorts	n	Age (mean, SD and range)	Female to male ratio
Sepsis patients	99	56.26 +/- 14<br>(19-83)	58/41
Control group	53	42.41 +/- 11.7<br>(26-76)	25/28
Pre-surgical group	19	59.29 +/- 11.94<br>(36-80)	8/11
Post-surgical group	11	59.16 +/- 12.11<br>(18-77)	6/5
