# Supplementary material for: Circulating miRNAs in sepsis—A network under attack: An in-silico prediction of the potential existence of miRNA sponges in sepsis
Source: PLoS One. 2017 Aug 18;12(8):e0183334. doi: 10.1371/journal.pone.0183334 (PMC5562310; doi:10.1371/journal.pone.0183334)
Supplement: S2 Table — (PDF) [file pone.0183334.s002.pdf]

distance	strength	color
from 0 to 6	very powerful connected	red
from >6 to 10	powerful connected	blue
from >10 to 20	medium connected	green
from >20 to 40	slightly connected	black
from >40 to 80	poor connected	black
above 80	very poor connected	black
