# Supplementary figures and images for: Circulating miRNAs in sepsis—A network under attack: An in-silico prediction of the potential existence of miRNA sponges in sepsis
Source: PLoS One. 2017 Aug 18;12(8):e0183334. doi: 10.1371/journal.pone.0183334 (PMC5562310; doi:10.1371/journal.pone.0183334)

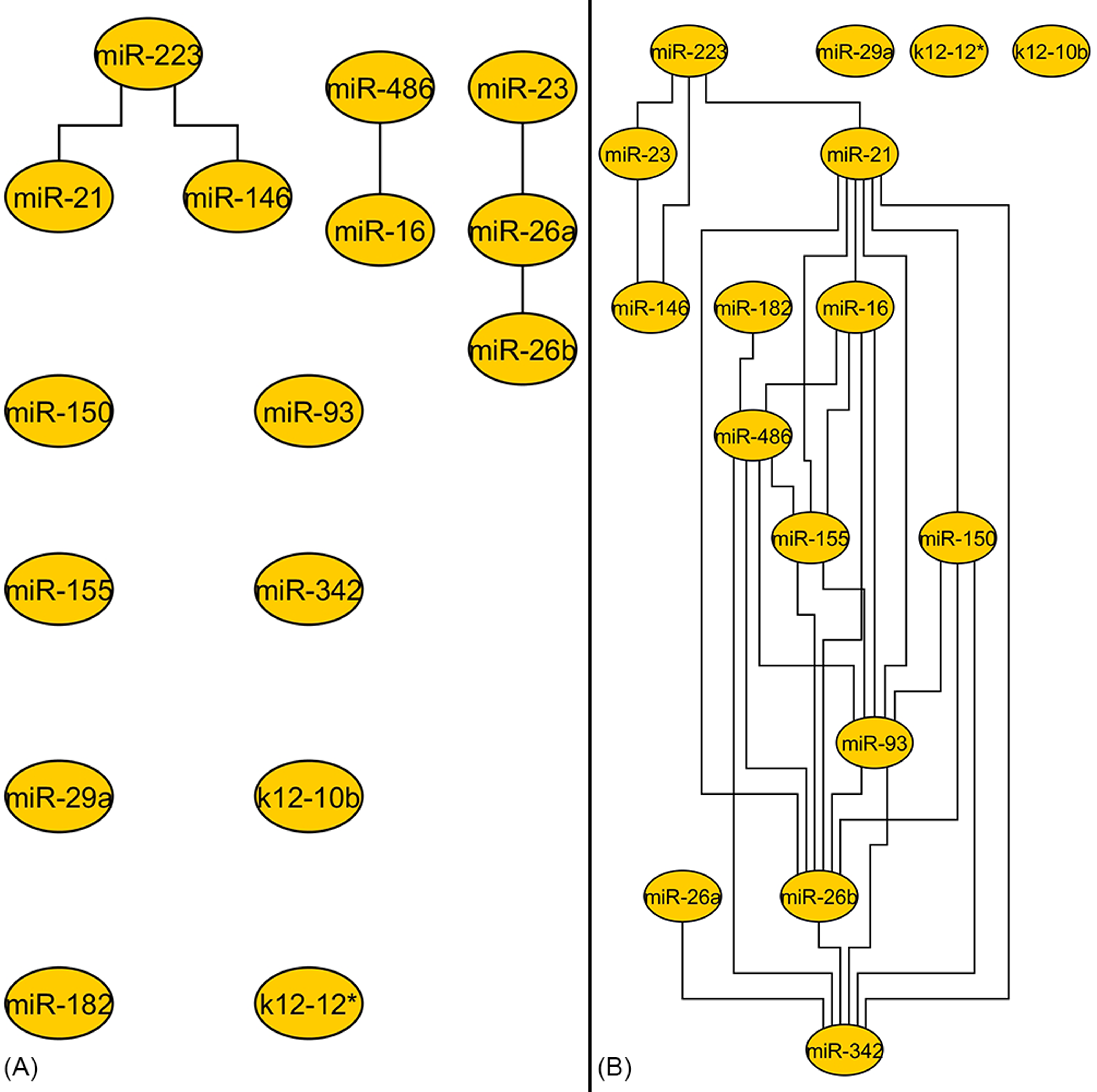

Supplement: S1 Fig — On the left side the pre-surgical miRNA network is represented (A) which contains only 5 edges, on the right side the postsurgical miRNA network is depicted (B), which contains 28 edges. After the surgical procedure the number of edges significantly increases from 5 to 28 (P = 0.0001), this is on increase of 82.14%. All the existing edges from the pre-surgical group are also present in the post-surgical group. (TIF) [file pone.0183334.s004.tif]

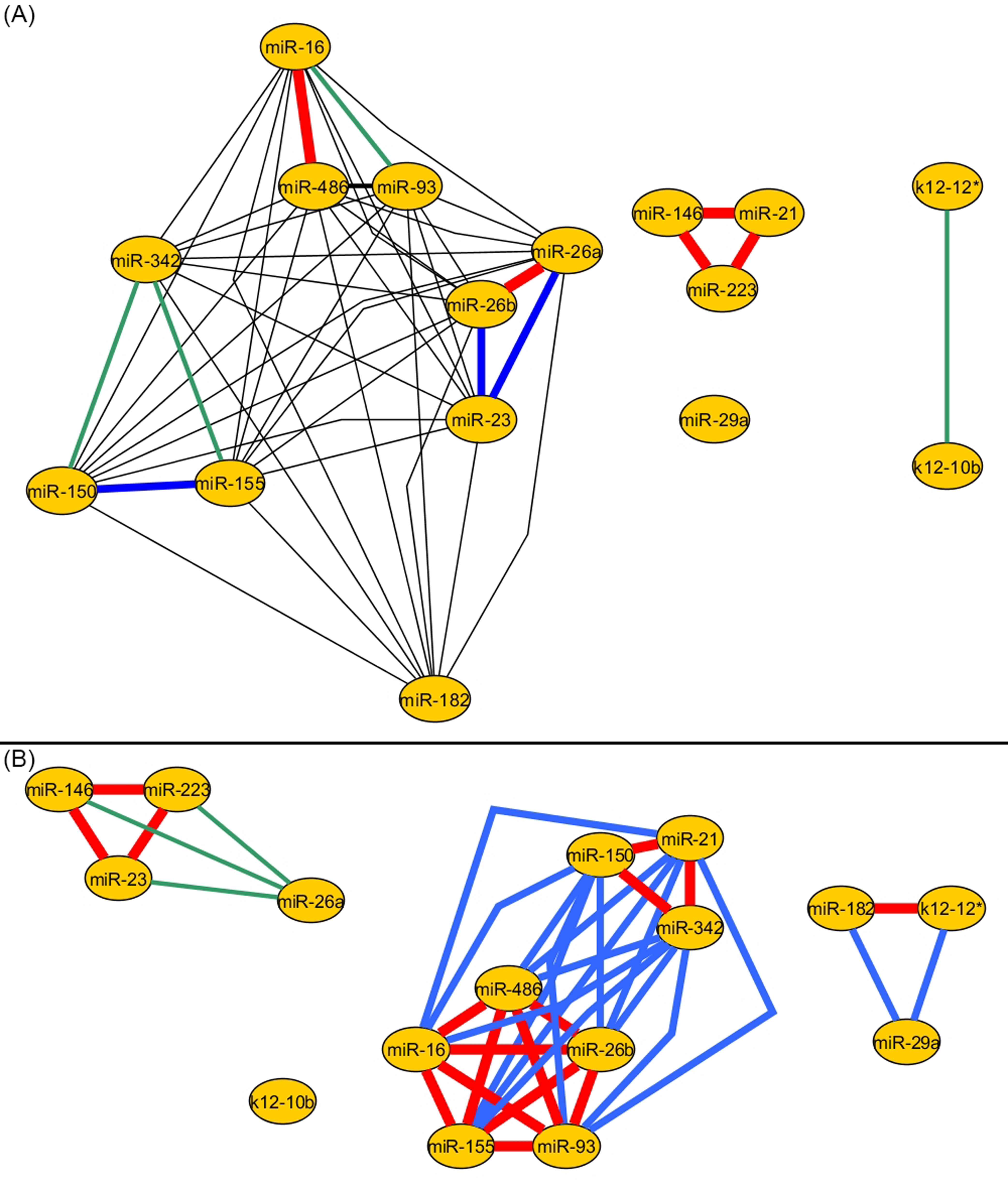

Supplement: S2 Fig — In the upper part of the figure the pre-surgical miRNA network is depicted (A), which is built out of 4 clusters and a total of 49 edges. In the lower part of the figure the post-surgical miRNA network is depicted (B), consisting of 4 clusters and 37 edges. In this case the number of edges decreases, from 49 to 37 (P = 0.0763 –not statistically significant), this is a decrease of 24.48%. This observation is similar to the data obtained for the control and septic patient networks, where the number of edges also decreases, but in the case of cluster analysis, much more meaningful is the distance between the edges, which clearly increases between the pre-surgical network and the post-surgical network. In the case of the control and septic patient network the distance decreases between the nodes of the control and septic patient miRNA networks. The total distance of the pre-surgical miRNA network is 1138 and the total distance of the post-surgical miRNA network is 188 (P = 0.0001). This observation is opposite to what we observed in the case of control and sepsis miRNA networks, where the distance decreases. (TIF) [file pone.0183334.s005.tif]

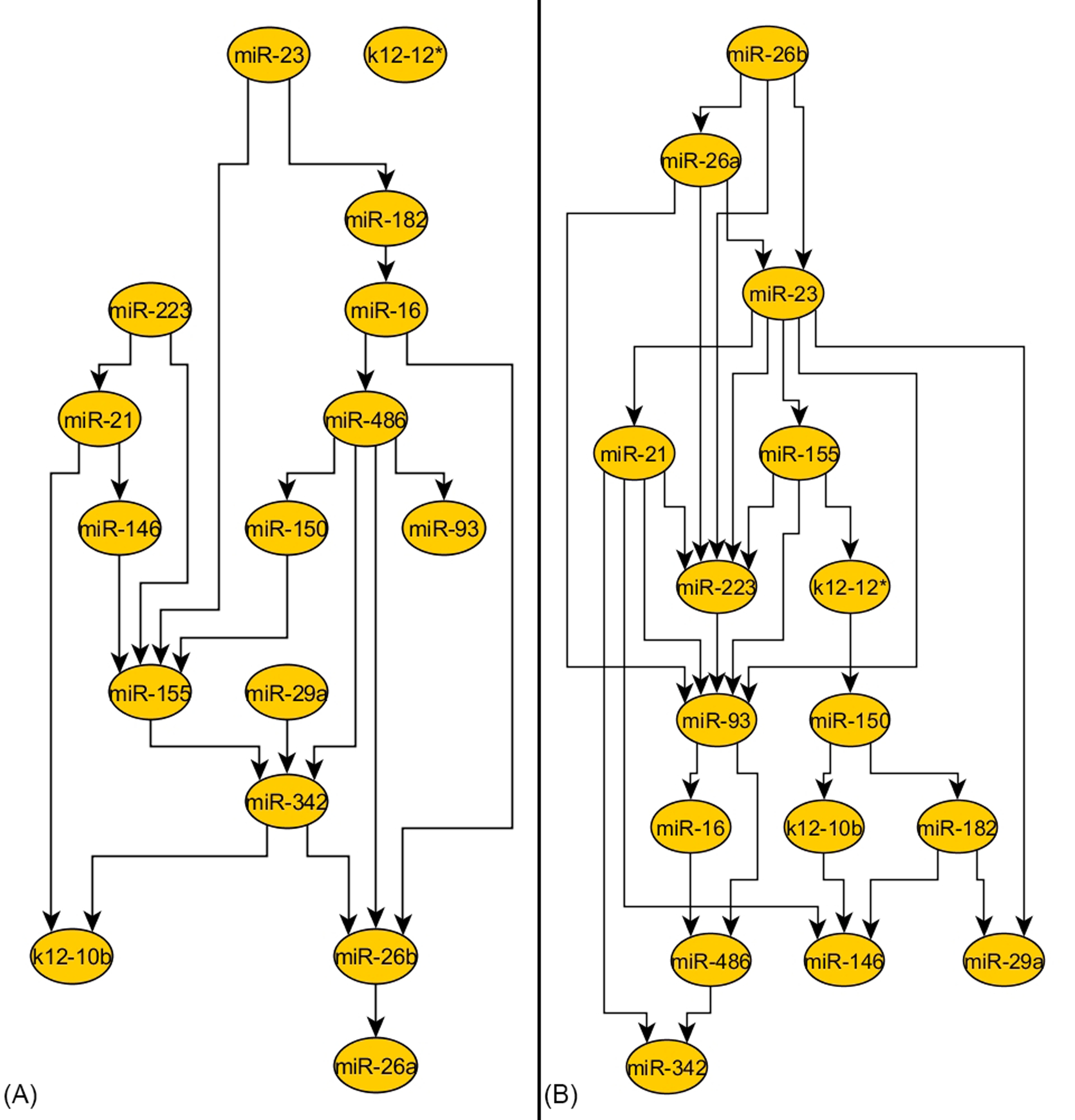

Supplement: S3 Fig — On the left side the pre-surgical miRNA network is represented (A), composed of 16 nodes and 16 edges, on the right side the postsurgical miRNA network is depicted (B) composed of 16 nodes and 29 edges. When using this method, the number of edges increases between the two networks, from 20 to 29, an increase of 31.03%, this difference is not statistically significant (P = 0.1999). This observation is opposite to what we observed in the case of control and sepsis miRNA networks built using the Bayesian method, where the number of edges decreases. (TIF) [file pone.0183334.s006.tif]
